# Supplementary material for: Ultrafast imaging of polariton propagation and interactions
Source: Nat Commun. 2023 Jun 30;14:3881. doi: 10.1038/s41467-023-39550-x (PMC10313693; doi:10.1038/s41467-023-39550-x)
Supplement: Supplementary file 3 — Description of Additional Supplementary Files [file 41467_2023_39550_MOESM3_ESM.pdf]

Description of Additional Supplementary Files

File name: Supplementary Movie 1

Description: Movie of exciton transport corresponding to data in Figure 1c of the main text.

File name: Supplementary Movie 2

Description: Movie of exciton-polariton transport corresponding to data in Figure 1d of the main text.
